# Root-Canal Filling

**Published:** 1903-03-15

**Authors:** B. F. Arrington

**Affiliations:** Goldsboro, N. C.


### ROOT-CANAL FILLING.

BY B. F. ARRINGTON, D.D.S., GOLDSBORO, N. C.

In the September issue of the *Digest* I notice a paper by Dr. Beck of Chicago, entitled, "Paraffine, an Ideal Material for the Filling of Root-canals," and in the closing paragraph of the discussion of the paper Dr. Beck says, "I do not claim originality in the use of paraffine, but if any one used it previously he failed to record it, as I can not find any record of its employment for root filling."

There was a period in the history of dentistry (forty or fifty years ago) when there were but few dental journals in existence. Then a dentist could easily keep informed of all papers and subjects of interest that were published but for fifteen or twenty years past journals have been so numerous that it is difficult to keep pace with them, consequently many of us loose much of valuable matter that is published, and can say as Dr. Beck, "I find no record of it."

If I could spare the time to look over some stored-away journals I think I could cite Dr. Beck several papers pertaining to the subject that were published as recently as twelve or fifteen years ago. About that period and later on I experimented considerably with paraffine in root-canal filling, but was not so favorably impressed with it as with beeswax, a material I have been using successfully for more than a quarter of a century.

Dr. Beck is moving on the right line for best results in root filling, and if, after thoroughly testing the merits of paraffine, he will accept the suggestion to drop it and substitute beeswax (best quality), and change his mode of conveying to cavity and manipulating the material in root-canals, he will possibly have reached as near the zenith of perfection in root-canal filling as is attainable.

For the benefit of any it may interest I will briefly state my practice in use of beeswax for root-canal filling. It will be well, however, to first state the claim for such an operation as I understand it, the object is to obliterate the vacuum, and to preclude if possible ingress of fluids or microbes. To do this effectually I have never in long years of experimenting found any material equal to beeswax. In properties it is all that can be desired. It is non-irritant, a non-conductor, and is non-destructible as a remedy in dental practice. Fire is the only element of destruction. No acid known to chemists nor alcohol in any way weakens its properties. If forced through the foramen at apex of canal no harmful result will follow. It can be easily and quickly applied and successfully manipulated in root-canals, and results will follow that are entirely satisfying. Before opening and treating the canals, shape and prepare the cavity in crown of tooth, wash carefully, using bulb-syringe, dry with bibulous paper, mop walls, with saturated solution of silver nitrate, and dry with hot air. Then venture into pulp chamber and root-canals, treat thoroughly, and prepare for filling as can be best accomplished, If there is no disease at apex nor any trace of septic odor it is safe to fill without delay. Have all things in readiness so that there may be no hitch or confusion after commencing operation.

Prepare against moisture, dry cavity and root-canals with bibulous paper and hot air, place a pellet of wax in pulp chamber and apply a heated burnisher, size and shape to suit. The melted wax will flow readily into canals and smallest crevices. After use of burnisher, to quickly melt and flow the wax, commence with drawn-temper root-canal pluggers, different sizes, using largest size first, then next size and so on, heating and re-heating at every application, adding wax if necessary. If the canals are very contracted follow use of pluggers with broaches, heating frequently to make sure of forcing wax to apex. I sometimes use delicate wisps of cotton fiber incorporated with the wax, especially when the foramen is large. Packing the fiber with heated instruments, the cotton soon becomes thoroughly incorporated with the wax and makes a secure apex safeguard. After the canals are filled, fill pulp chamber after same order, with more of cotton fiber, using considerable force, then with a spoon-shaped excavator remove any fragment of wax from walls of cavity, re-apply nitrate of silver solution to the walls, and dry with hot air. Base with sheet lead, thickness to suit, or gutta-percha, and fill as judgment directs. Such has been my practice for many years and almost universal satisfaction to patients and operator has been the result.—*Digest.*

